# A systematic review of ecological momentary assessment studies of appetite and affect in the experience of temptations and lapses during weight loss dieting

**DOI:** 10.1111/obr.13596

**Published:** 2023-07-02

**Authors:** Mark Randle, Amy L. Ahern, Emma Boyland, Paul Christiansen, Jason C. G. Halford, Jack Stevenson‐Smith, Carl Roberts

**Affiliations:** ^1^ Cardiff University Brain Research Imaging Centre Cardiff UK; ^2^ MRC Epidemiology Unit University of Cambridge Cambridge UK; ^3^ Department of Psychology University of Liverpool Liverpool UK; ^4^ School of Psychology University of Leeds Leeds UK

**Keywords:** dietary temptation, ecological momentary assessment, lapse, obesity

## Abstract

Dietary temptations and lapses challenge control over eating and act as barriers toward successful weight loss. These are difficult to assess in laboratory settings or with retrospective measures as they occur momentarily and driven by the current environment. A better understanding of how these experiences unfold within real‐world dieting attempts could help inform strategies to increase the capacity to cope with the changes in appetitive and affective factors that surround these experiences. We performed a narrative synthesis on the empirical evidence of appetitive and affective outcomes measured using ecological momentary assessment (EMA) during dieting in individuals with obesity and their association with dietary temptations and lapses. A search of three databases (Scopus, Medline, and PsycInfo) identified 10 studies. Within‐person changes in appetite and affect accompany temptations and lapses and are observable in the moments precipitating a lapse. Lapsing in response to these may be mediated through the strength of a temptation. Negative abstinence‐violation effects occur following a lapse, which negatively impact self‐attitudes. Engagement in coping strategies during temptations is effective for preventing lapses. These findings indicate that monitoring changes in sensations during dieting could help identify the crucial moments when coping strategies are most effective for aiding with dietary adherence.

## 1 INTRODUCTION

Dieting is the self‐imposed restriction over eating behavior to achieve an energy deficit. Adhering to healthy and attainable dietary regimens is considered the most important factor for initial weight loss.^1^ Unfortunately, most attempts are unsuccessful, as many individuals are unable to achieve and maintain modest losses (i.e., between 5% and 10% loss maintained over a year).^2^ One major challenge dieters face is that appetite regulation appears to be asymmetric.^3^ There are strong regulatory systems within the body to defend against energy deprivation (regardless of current weight status or fat stores), while systems that defend against weight gain are permissive of excess energy and are easily overridden by the hedonic aspects of food.^4^ These effects are greater in individuals with overweight and obesity^5^ making those who would benefit the most from weight loss also experience the greatest challenges in achieving and maintaining it.

The changes that occur as a result of maintaining a negative energy balance overwhelm control over eating behavior, which contributes to the low rate of dietary compliance. Heightened sensations of appetite (e.g., hunger and cravings) and negative mood being the most common reasons provided for unsuccessful dieting attempts.^6^, ^7^, ^8^ Currently, very little is known about the lived‐experiences common in dieters which constitute as the real‐world barriers for successful dietary adherence, that is, temptations and lapses.^9^

Dietary temptations are the sudden urge to eat, which brings a person to the brink of breaking a diet. They are the output of momentary reward‐based evaluations of an environmental food‐related stimuli that has previously been paired with consumption.^10^ These food cues activate reward‐circuitry and trigger appetitive motivational processes (e.g., food cravings) that orientate behavior toward consumption of the reward.^10^ Temptation strength is a common reason for consuming unhealthy snacks^11^ and mediates the relationship between implicit evaluations of unhealthy snack food and subsequent intake.^12^ The abundance of environmental food cues present in daily lives could lead to the persistent temptation to overindulge.^10^

Dietary lapses are incidences where dietary goals have been violated (e.g., overate and ate a forbidden food), putting weight loss or maintenance at risk.^9^ Previous investigations into dietary lapses have relied on structured interviews following weight loss or focused on retrospective recall of a specific moment of lapsing. These reports indicate that lapses are precipitated by temptations,^10^ and driven by high levels of hunger, cravings, negative affect, being in the presence of food‐related cues, and social situations.^13^, ^14^

An additional challenge in dieting is how individuals respond to lapses. Those unsuccessful in weight loss report greater negative abstinence‐violation effects (e.g., feelings that the diet will not be successful), which detrimentally impact self‐attitudes (e.g., self‐efficacy, self‐regard, and self‐compassion) surrounding their ability to successfully maintain the diet.^15^

A high level of adherence to a diet is a crucial factor for successful weight loss.^16^ Those who are unsuccessful at achieving and maintaining weight loss demonstrate ineffective coping strategies and self‐regulatory abilities to manage temptations.^12^, ^13^ Developing strategies to increase the capacity to cope with heightened responses during these experiences is crucial for successful weight loss.

Our understanding of how these act in the real‐world during experiences of dietary temptations and lapses is limited. Appetite and affect are transient and environmentally driven,^17^ particularly during a negative energy balance. Laboratory and retrospective approaches fail to capture the environmental or temporal influences meaning these effects on eating behavior can only be alluded to.

Ecological momentary assessment (EMA) addresses this by directly focusing on a person's current feelings, behaviors, and surrounding environmental influences. Measures are taken repeatedly within their natural environment to capture how experiences fluctuate across time and context.^18^ Assessments are employed based on the feature of interest, that is, using event assessments to measure discrete behaviors or events such as experiencing a temptation or lapse; or applying random sampling to characterize daily experiences and how levels fluctuate throughout the day.

One analysis approach is to collapse observations across assessment types (e.g., events such as temptations and lapses; random assessments throughout the day) so that outcomes can be contrast between different experiences.^19^ The focus of this approach is to evaluate how outcomes differ between experiences. For example, how are sensations of hunger different when experiencing a temptation compared to a random moment where no temptation or lapse has recently taken place? Another approach is to ask whether participants have lapsed since their last assessment. Previous ratings are used to prospectively predict how changes influence lapsing. The focus of this analysis approach is in the moments leading up to an event.

The benefit of using EMA is that frequently assessing experiences within their natural environment can generate insight into the temporal dynamics of the factors influential to dietary adherence.^17^ A review on the momentary influences of temptations and lapses would provide a better understanding of how these experiences unfold within real‐world dieting attempts. This would help inform strategies to cope with changes, which underlie these experiences.

The aim of this investigation was to systematically review studies that have used EMA to assess subjective sensations of appetite and affect in adults living with overweight or obesity who are engaging in dieting attempts to synthesize empirical evidence on how these sensations are associated with experiences of temptations and dietary lapses. First, we contrast sensations of appetite and affect in the presence (or absence) of dietary temptations or dietary lapses. Second, we evaluated how within‐person changes prospectively predicted dietary lapses. Finally, given previous data on the importance of coping strategies and abstinence violation effects in studies of temptations and lapses, we synthesized evidence for these factors in EMA studies, which took either a contrast or prospective approach.

## 2 METHODS

### 2.1 Information sources and search strategy

The current systematic review is reported in accordance with the Preferred Reporting Items for Systematic Reviews and Meta‐analyses (PRISMA) and was preregistered on PROSPERO (crd.york.ac.uk/prospero/display_record.php?ID=CRD42018115796). Three electronic databases: Scopus, MEDLINE, and PsycInfo were searched in September 2019 using the following string: (Obes* OR Overweight) AND (“Ecological momentary” OR “Experience sampling” OR “Intensive longitudinal” OR “Daily diary”) AND (Diet* OR Weight OR Energy OR Calorie) with no date limitations. Searches were updated in January 2023 by reperforming searches with a date range from 2019 to present. A manual searches of reference sections of included articles was also performed.

### 2.2 Eligibility criteria

Eligible studies had to meet the following criteria:

#### 2.2.1 Participants

Studies were eligible if they investigated human participants between the ages of 18–65 years living with overweight or obesity (BMI ≥ 25 kg/m^2^). Investigations that exclusively recruited populations with diagnosed diseases, conditions, or disorders that may impact appetite or eating behavior (e.g., diabetes) were excluded. Investigations that included patients of bariatric surgery or with history of any eating disorders were also excluded.

#### 2.2.2 Interventions

Studies were eligible if they employed any form of dieting (e.g., structured weight loss intervention or self‐directed attempts) for any period of time.

#### 2.2.3 Outcome measures

Studies were eligible if they used EMA methods to measure any subjective ratings of appetite and affect (inclusive of abstinence‐violation effects and self‐attitudes) or engagement in coping strategies using any analytical approach to assess experiences of temptations and lapses. Outcome measures were not included in search strings to avoid omission of any relevant studies through differences in terminology but were screened during full text review (see Table 1 for description of all outcome measures).

**TABLE 1:** Summary table of characteristics and findings of all included studies in the systematic review and meta‐analyses.

Author	Design	Domain	Outcome measures	EMA assessment and analysis strategy	Results
Carels et al.^20^	*N* = 30 (73% F, 9% minority ethnicities, mean age (SD) = 19.88 (4.4), mean BMI (SD) = 29.2(3.9)). 1 week of EMA	Appetite and affect	5‐point Likert scale (*not at all* to *extremely*) assessing hunger, positive and negative mood	RA, TA and LA Contrast of assessment types	Greater hunger and negative mood on TA and LA. Less fullness on TA and less satisfied on LA.
	Self‐guided dieting attempts (12% weight loss maintenance; 88% weight loss)	Abstinence‐violation effects	Responses from four 5‐point Likert scales (*not at all* to *extremely*) summed to produce separate negative and positive AV effects	RA, TA, and LA Contrast of assessment types	Greater negative AV on TA and LA. TA greater positive AV compared to LA.
Carels et al.^21^	*N* = 37 (100% F, 94.5% Caucasian, mean age (SD) = 55.4 (7.9), mean BMI (SD) = 29.2(5.5)). 1 week of EMA	Appetite and affect	5‐point Likert Scale (*not at all* to *extremely*) assessing hunger, positive and negative mood	RA, TA and LA Contrast of assessment types	Greater positive and negative mood on LA compared to RA
	Final week of weight loss intervention	Abstinence‐violation effects	Responses from four 5‐point Likert scales (*not at all* to *extremely*) summed to produce separate negative and positive AV effects	RA, TA and LA Contrast of assessment types	Greater negative AV effects on LA
		Coping strategies	Response from 14 5‐point Likert scales (*not at all* to *extremely*) summed to produce coping strategies score	TA and LA Contrast of assessment types	Coping more strongly associated with TA than LA
Chwyl et al.^22^	*N* = 107 (83.5% F, 69.7% Caucasian, mean age (SD) = 52.36, mean BMI (SD) = 35.35 (5.28)). Secondary analyses of Forman et al.^23^ 3 weeks of EMA Mid‐treatment (6 months) of a 12‐month structured behavioral weight loss program.	Coping strategies	Participants responded to a multiple response question inquiring about engagement in various activities (eating, chores, cooking, indoor hobby, outdoor recreation or exercise, socializing, spiritual activity, prayer of meditation, TV/social media, work or school, other) since the last survey. These were selected based on coping strategies used in Carels et al.^21^	RA and LA Prospective analyses of lapses	Engaging in chores, cooking, spiritual activity, or work/school decreased the likelihood of a lapse being reported at the current assessment. Socializing increased the likelihood of a lapse being reported at the current assessment Cooking and an indoor hobby increased the odds that a lapse would be reported at the next assessment
Crochiere et al.^24^	*N =* 159 (83.2% F, 72% Caucasian, mean age (SD) = 47.79 (12.65) mean BMI (SD) = 36.5 (12.72)). Secondary analyses of Forman et al.^25^ and Forman et al.^26^ combined dataset 2 weeks of EMA 2‐week calorie reduced diet delivered through the weight watchers app	Appetite and affect	5‐point Likert Scale (*not at all* to *extremely*) measuring hunger, tiredness, boredom, mood and food cravings. Mood consisted of (responses from “Especially good mood” to “Intensely stressed/upset”)	RA and LA Prospective analyses of lapses	Within person levels of tiredness, food cravings, associated with lapsing at the next assessment
Forman et al.^9^	*N* = 189 (82% F, 70.9% Caucasian, mean age (SD) = 51.81 (9.76), mean BMI (SD) = 36.93 (5.8)). 2 weeks in first 2 weeks, 1 week at 6 months, and 1 week at 12 months of EMA 12‐month behavioral weight loss program	Appetite and affect	5‐point Likert Scale (*not at all* to *extremely*) measuring hunger, and summed responses from 7 scales to produce negative mood	RA and LA Prospective analyses of lapses	Hunger and feelings of deprivation associated with lapsing at the next assessment
Goldstein et al.^27^	Secondary analysis of Forman et al.^9^	Individual differences in lapse triggers	Lapse type (eating an unintended food, eating at an unplanned time, eating a larger portion than intended), appetite, affect, and context measures	RA and LA Prospective analyses of lapses	Within‐person increases in hunger and deprivation, and decreases in irritability associated with eating an unintended food at the next assessment Within person increases in stress and boredom associated with eating at an unplanned time at the next assessment Within person increases in deprivation and hunger associated with eating a larger portion at the next assessment
McKee et al.^28^	*N* = 80 (80% F, 80% White, 12% Asian, 6% Black, 2% other Ethnic origin, mean age (SD) = 41.21 (15.60), mean BMI (SD) = 30.78 (7.26)). 1 week of EMA Recruited from local weight loss groups (45%) and self‐attempted dieting (55%)	Appetite, affect, and coping strategies	Six 7‐point Likert scales (*not at all* to *very much*) assessing hunger, temptation strength, 2 separate summed responses from 2 scales to produce negative mood and coping	TA and LA Contrast of assessment types	Coping was negatively associated with lapse, and negatively predicted temptation strength while positively predicting self‐efficacy for future temptations. Coping, stress, and hunger were indirectly associated with lapses via temptation strength.
Sala et al.^29^	*N =* 126 (83.3% F, 69% Caucasian, mean age (SD) = 51.8 (11.8), mean BMI (SD) = 35.5 (5.1)). 1 week of EMA Prior to randomization in a behavioral weight loss intervention	Appetite	One 5‐point Likert scale (*not at all* to *strongly*) assessing cravings	RA and LA Prospective analyses of lapses	Higher levels of within‐person levels of cravings was associated with a higher likelihood of reporting a lapse at the current assessment
Schumacher et al.^30^	Secondary analysis of Forman et al.^9^	Self‐attitudes and lapse frequency	4‐point Likert Scales (*not at all critical* to *extremely critical*) assessing self‐criticism, self‐forgiveness, self‐regard and self‐efficacy	RA and LA Prospective analyses of lapses	Lower within‐person self‐criticism predicted same‐day lapse.
Thøgersen‐Ntoumani et al.^31^	*N =* 56 (92.86% F, 78.60% Caucasian, mean age (SD) = 34.88 (13.93), mean BMI (SD) = 32.50 (6.88)). Two weeks of EMA Self‐guided dieting attempts	Self‐attitudes and AV effects	Responses from three 5‐point Likert scales (*almost never* to *almost always*) measuring self‐compassion. Responses from four 11‐point Likert scales (0% to 100%) measuring self‐efficacy Responses from two 6‐point Likert scales (*strongly disagree* to *strongly agree*) measuring negative reactions to lapses Responses from fifteen 5‐point Likert scales (*not feeling this way at all* to *feeling this way very strongly*) measuring self‐conscious emotions (aggregated into measures of guilt and shame) Responses from two 7‐point Likert scales (*strongly disagree* to *strongly agree*) measuring intention to continue dieting	RA and LA Consequences of lapses	High within person levels of self‐compassion was associated with greater intentions to continue dieting, self‐efficacy, and less negative reactions following dietary lapses. Higher within‐person self‐compassion was related to lower levels of shame and guilt following a lapse. Higher within‐person levels of guilt was related to lower intentions, self‐efficacy, and negative reactions following a lapse. Guilt mediated the relation between self‐compassion and intentions, self‐efficacy, and negative reactions following dietary lapses

Abbreviations: AV, abstinence violation; EMA, ecological momentary assessment; LA, lapse assessment; RA, random assessment; TA, temptation assessment.

### 2.3 Data search and extraction

#### 2.3.1 Article selection and data extraction

One author (MR) performed the formal database searches and supplementary searches. Two authors (MR and JSS) assessed articles for inclusion. Data were extracted independently by two coders and cross‐checked with disagreements being resolved through discussion.

The following data were extracted for each study: Number of participants, average number of within person assessments, sex (% female), age, ethnicity, BMI, duration of EMA procedure, and a description of the type of dieting used. Finally, we extracted the type of analysis approach: Contrasts of assessment strategies to compare outcome measures in the presence and absence of temptations and lapses; the effect of within‐person changes in outcome measures on the likelihood that a lapse is reported at the current or next available assessment.

## 3 RESULTS

### 3.1 Study selection (Figure 1)

**FIGURE 1:**
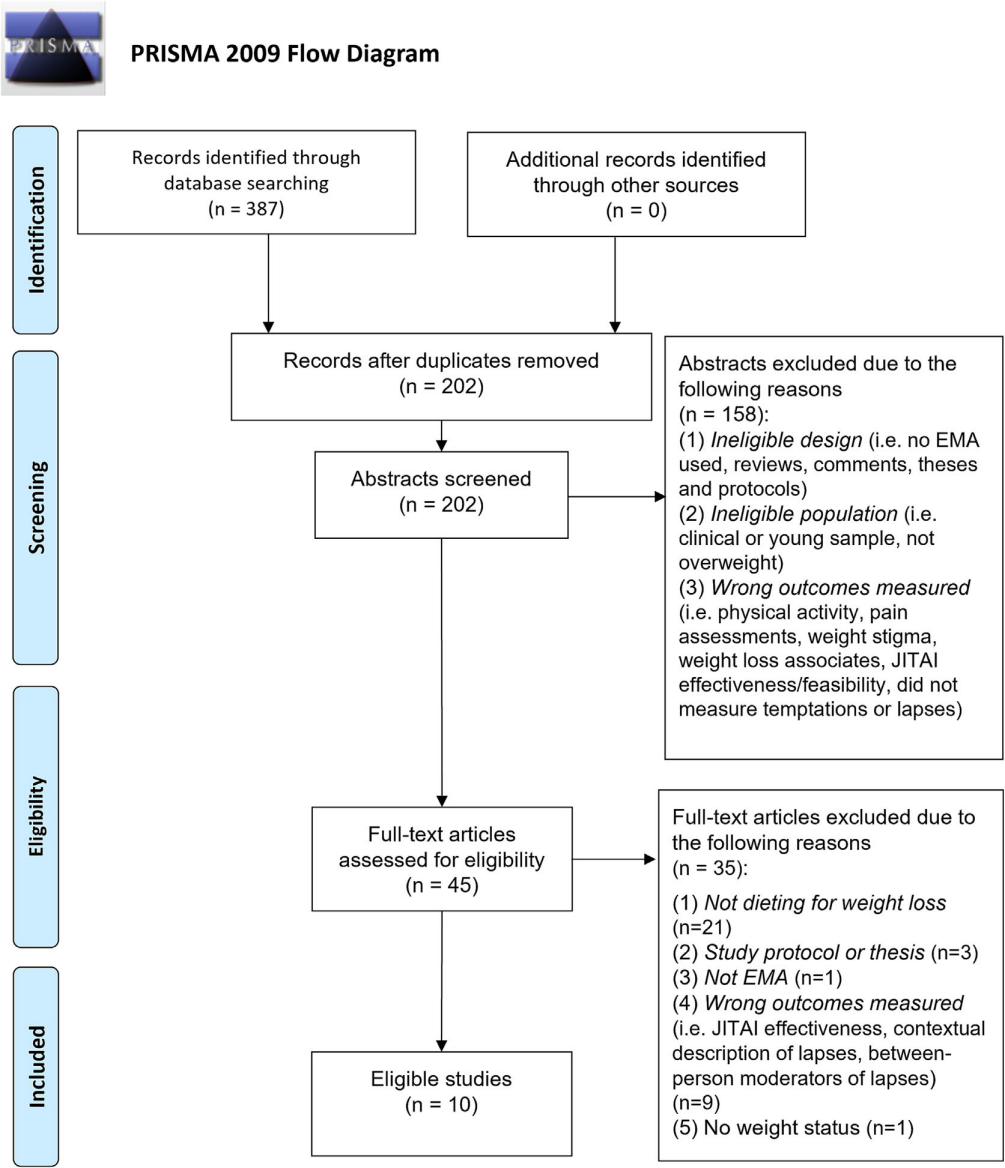
PRISMA flow diagram for study identification and inclusion.

The search strategy identified 131 studies using Medline, 117 using PsycInfo, and 139 using Scopus. Updated searches yielded four additional studies for inclusion.^22^, ^24^, ^29^, ^31^ Manual searches of reference sections did not reveal any additional papers. One hundred eighty‐five duplicates were removed leaving 202 papers for initial review. After screening titles and abstracts, 45 articles remained for full text review. A further 35 were excluded following full text review (see Figure 1 for reasons of exclusion) leaving a total of 10 papers.^9^, ^20^, ^21^, ^22^, ^24^, ^27^, ^28^, ^29^, ^30^, ^31^

### 3.2 Characteristics of included studies

Characteristics of included studies are displayed in Table 1. The mean age and BMI of participants from included studies was 44.39 years and 33.25 kg/m^2^, respectively, and the mean proportion of females was 84.73%.

Four studies^20^, ^21^, ^28^, ^29^ employed 1‐week long EMA testing procedures, two studies employed a 2‐week long testing procedure,^24^, ^31^ and one study employed a 3‐week EMA testing period.^22^ One study took place over a 12‐month period and conducted 2 weeks of EMA in the first 2 weeks, 1 week at 6‐month, and 1 week at 12‐month of the intervention.^9^ Two^27^, ^30^ were a secondary analyses of the aforementioned 12‐month study.^9^

Two primary investigations^9^, ^21^ were conducted during a structured behavioral weight loss intervention. One study^22^ was mid‐treatment of a 12‐month structured weight loss intervention.^23^ One study took place in a baseline phase of a trial prior to randomization to a structured weight loss intervention.^29^ Three studies^20^, ^28^, ^31^ examined self‐guided dieting attempts including both dieting for weight loss and for weight loss maintenance. One study^24^ combined data from two studies with identical protocols, which used the Weight Watchers mobile program to prescribe an energy reduced diet.^25^, ^26^

### 3.3 Quality assessments

We used two forms of quality assessment (see S.1.1 in the Supporting Information for a description). We assessed the quality of reporting with an established Strengthening the Reporting of Observational Studies in Epidemiology (STROBE) checklist adapted for reporting EMA studies (CREMAS).^35^ We assessed the quality of methodological components of the included studies using the Newcastle‐Ottawa cohort scale (NOS) adapted for cross sectional studies.^36^ We modified this tool to assess the qualities of EMA investigations.

These tools were used to assess the quality of individual studies for descriptive purposes only and did not inform data synthesis.

### 3.4 Narrative synthesis

#### 3.4.1 Aim 1: Contrasting appetite and affect in the presence or absence of dietary temptations and lapses

**FIGURE 2:**
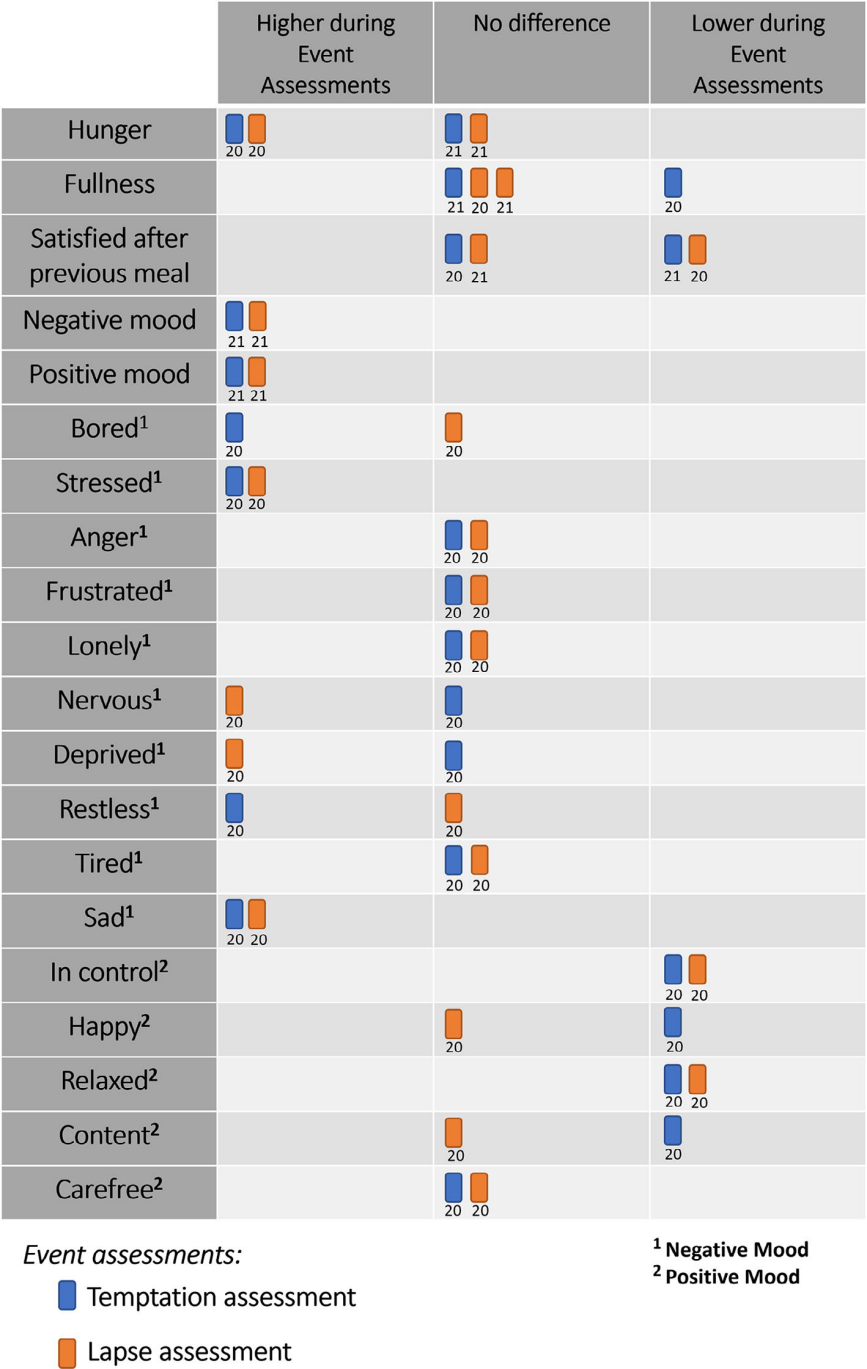
Harvest plot of appetite and affect outcomes for studies that contrast event assessments (temptation/lapse assessments) with random assessments. Blue and orange boxes represent temptation and lapses respectively. If a box falls on the left or right, outcomes were higher or lower during event assessments, respectively. Boxes listed in the middle column denote no difference between assessment types. Numbers under boxes denote the study reference.

Two studies from Carel et al.^20^, ^21^ contrasted appetitive and affective outcomes for temptation assessments versus random assessments and lapse assessments versus random assessments. See Figure 2 for a harvest plot illustrating these findings.

Carels et al.^20^ found hunger was higher during temptations and lapses, fullness was lower during temptations (but not lapses), and satisfaction with a previous meal was lower during lapses (but not temptations). Levels of boredom, stress, restlessness, and sadness were raised during temptations, whereas feeling in control, happiness, relaxedness, and contentedness were lower during temptations. Levels of stress, deprivation, nervousness, and sadness were raised during lapses, whereas levels of feeling in control and relaxedness were lower during lapses.

Another study by Carels et al.^21^ found no evidence that hunger or fullness was different during temptations or lapses. There was also no evidence that satisfaction with a previous meal was different during temptations, but levels were found to be lower during lapses. Finally, they found that overall positive and negative mood scores were higher during both temptations and lapses.

McKee et al.^28^ contrasted outcomes between temptations that lead to a lapse with temptations that did not lead to a lapse. They reported an indirect relationship where higher levels of stress and hunger predicted temptation strength and this relationship influenced the likelihood that a temptation would lead to a lapse.

Taken together, these studies demonstrate that experiences of dietary temptations and lapses generally occur in the context of heightened appetite and affect. These responses are influential in the strength of temptations and risk of subsequent of lapsing. However, caution must be advised as there is a lack of overall evidence substantiating these claims.

#### 3.4.2 Aim 2: How changes in levels of appetite and affect prospectively predict dietary lapses

Forman et al.^9^ assessed whether appetite and affect prospectively predicted a lapse on the next available assessment. They found that increases in hunger and deprivation predicted a lapsing, but changes in all individual negative mood ratings (i.e., sadness, loneliness, boredom, stress, anger, and tiredness) did not predict lapsing.

A secondary analysis of this data characterized lapses into distinct subtypes (i.e., eating at an unintended time, eating an unintended food, and consuming a larger portion than intended) and investigated if increases in factors were prospectively predictive of different types of lapsing.^27^ They found that hunger, deprivation, and stress were predictive of all lapse types. Boredom and irritability were predictive of eating an unintended food. Boredom was also predictive of eating at an unintended time.

A similar study by Crochiere et al.^24^ found that increases in tiredness and cravings was associated with reporting a lapse at the next assessment, but levels of hunger, boredom and overall mood were not.

Sala et al.^29^ assessed whether increases in levels of food cravings prospectively predicted a lapse on the current and next available assessment. They found that increases in cravings were associated with reporting a lapse on the current assessment but not on the next available assessment.

**FIGURE 3:**
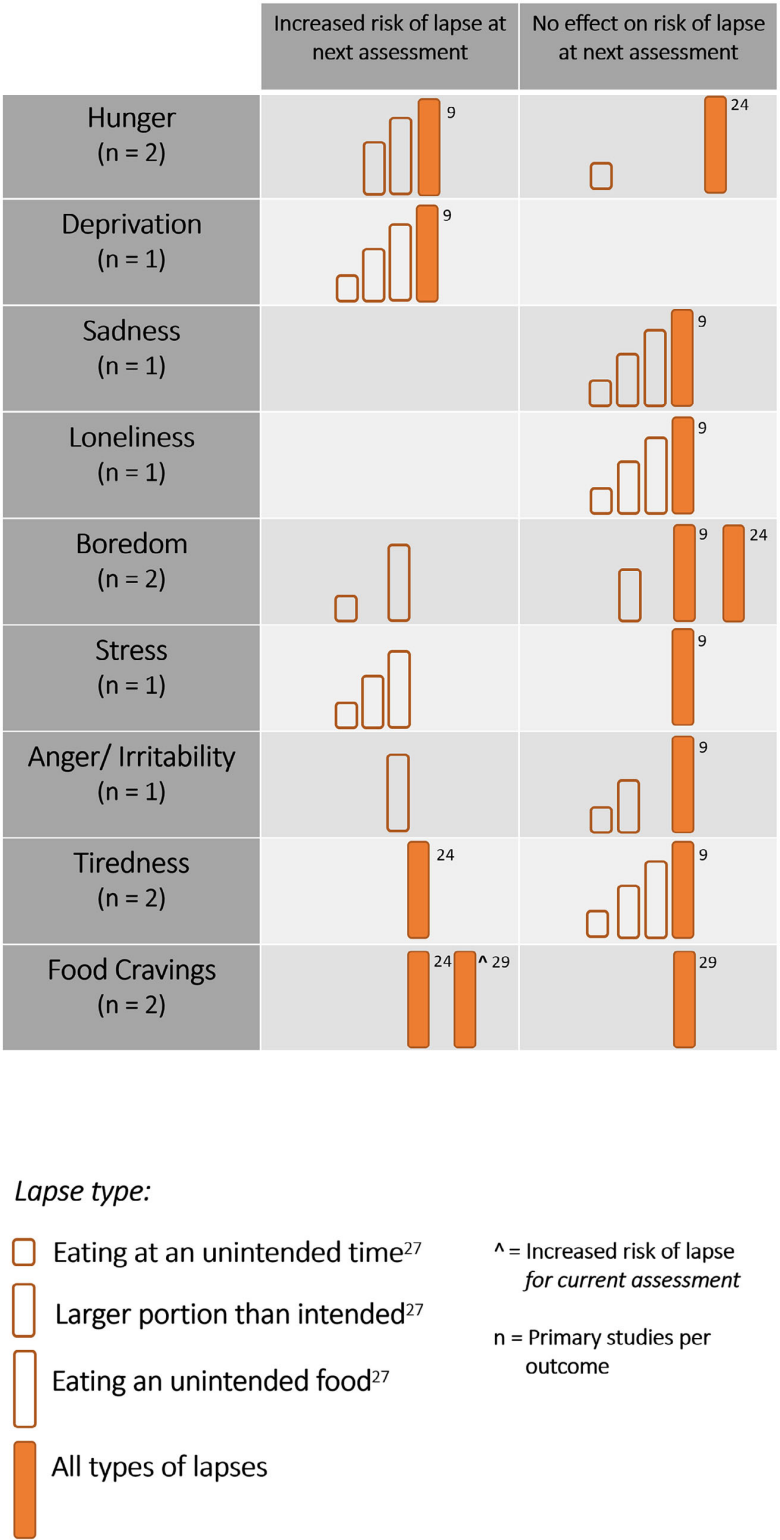
Harvest plot of within person triggers of a lapse at the next assessment for appetitive and affective outcomes. Evidence was found for an increased change that an outcome is associated with an increased likelihood of reporting a lapse at the next available assessment if a box falls in the left column. Boxes on the right column denote studies that found no effect. Size and fill of boxes represent lapse types. Smaller boxes with no fill represent effects for lapse subtypes from Goldstein et al.^27^ Large boxes with orange fill represent findings from a primary study, the study reference is denoted by the number next to each box.

See Figure 3 for a harvest plot illustrating the findings from the above studies.

Taken together, these studies demonstrate that a large array of appetitive and affective factors have been investigated in the context of prospectively predicting a lapse but currently lack a substantive amount of evidence. Changes may be influenced by situational (e.g., different types of lapses) and time‐based (e.g., lapse being reported on the current or next available assessment) factors.

#### 3.4.3 Aim 3: Synthesis of the evidence for abstinence violation effects and self‐attitudes during temptations and lapses

**FIGURE 4:**
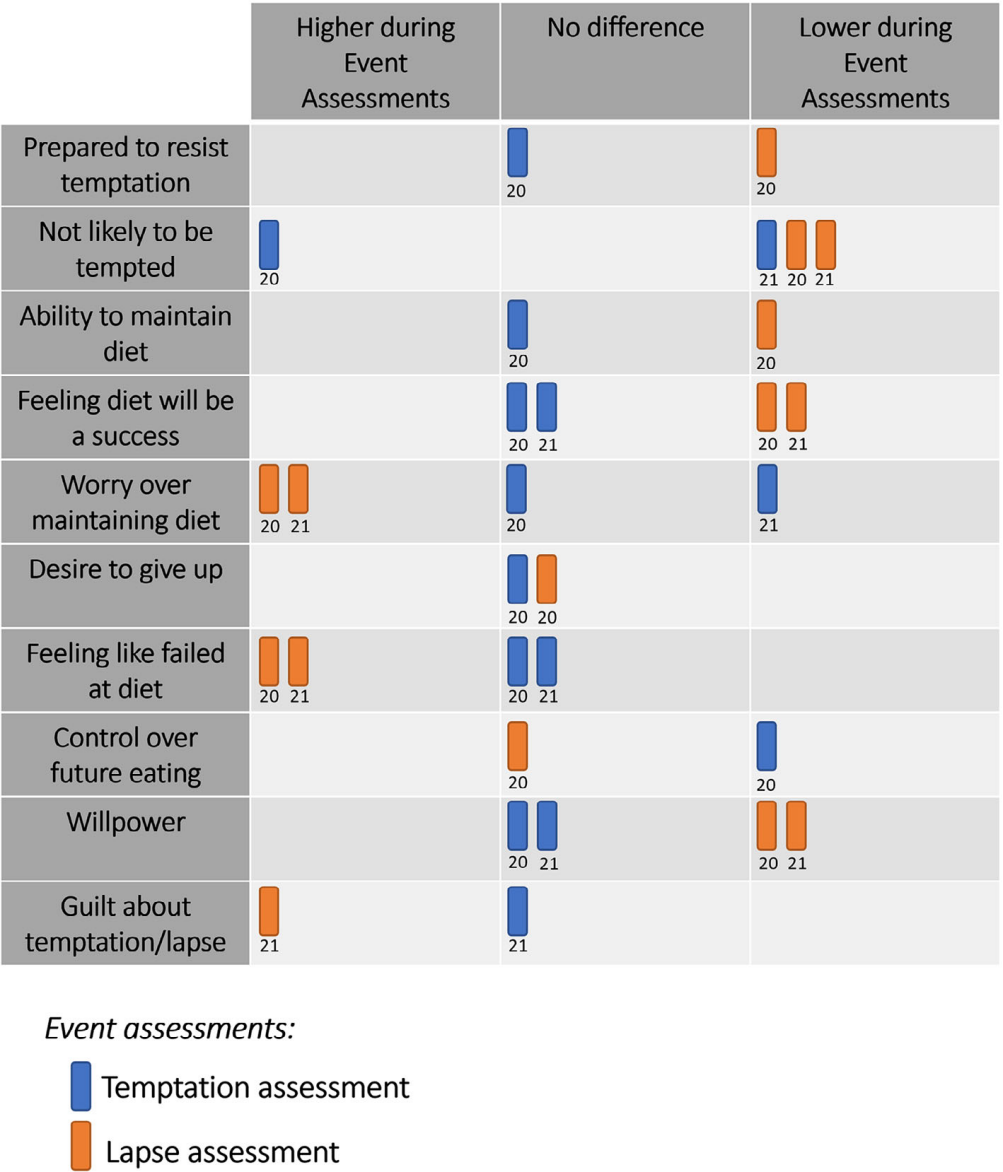
Harvest plot of abstinence‐violation outcomes for studies that contrast event assessments (temptation/lapse assessment) with random assessments. Blue and orange boxes represent temptation and lapses, respectively. If a box falls on the left or right, outcomes were higher or lower during event assessments, respectively. Boxes listed in the middle column denote no difference between assessment types. Numbers under boxes denote the study reference.

Two studies by Carel et al.^20^, ^21^ examined the presence of abstinence violation effects during temptations and lapses. They contrasted ratings on individual measures for temptation assessments versus random assessments and lapse assessments versus random assessments. See Figure 4 for a harvest plot illustrating findings from these studies.

They found that negative feelings about dieting efforts, particularly feelings of guilt, worry, and failure were higher during lapses (but not during temptations). Positive feelings about future diet success were lower during lapses (but no evidence for differences during temptations).

A secondary analyses^30^ of Forman et al.^9^ investigated whether self‐attitudes (i.e., self‐forgiveness, self‐criticism, self‐regard, and self‐efficacy) following a lapse were associated with experiencing another lapse on the same day. They found that decreased levels of self‐criticism predicted a greater chance of reporting another lapse reported on the same day, but changes in self‐forgiveness, self‐regard, and self‐efficacy were not.

Another study by Thøgersen‐Ntoumani et al.^31^ investigated the inter‐relationships between self‐attitudes (e.g., self‐efficacy, self‐compassion, and self‐criticism), intentions to diet, negative reactions, and feelings of guilt and shame after a lapse. They found that increases in self‐compassion was associated with greater intentions to continue dieting and levels of self‐efficacy. Self‐compassion was also associated with lower levels of negative reactions, shame, and guilt. Increased levels of guilt were related to lower levels of diet intentions, self‐efficacy, and negative reactions to a lapse. Guilt was found to mediate the relationship between self‐compassion and these variables (i.e., in moments when self‐compassion is high, intentions to continue dieting is also high which was mediated through lower levels of guilt). Feelings of shame did not predict diet intentions, self‐efficacy, or negative reactions following a lapse.

These few studies suggest that abstinence‐violation effects occur following lapses but are mostly absent from experiences of temptations. A negative reaction to lapsing can impact factors related to self‐attitudes, possibly through increased levels of guilt. These reactions may be detrimental to future dietary adherence, though some degree of self‐criticism following a lapse may be critical for preventing additional lapses on the same day.

#### 3.4.4 Aim 3: Synthesis of the evidence for engagement with coping strategies during temptations and lapses

Carels et al.^21^ examined the use of various coping strategies (e.g., removing oneself from the situation, distraction techniques, and encouragement from a friend) and contrast outcomes between temptation assessments vs. lapse assessments. They found that use of coping strategies were greater during temptations relative to lapses.

McKee et al.^28^ examined two strategies for coping with temptation (long‐term thinking of weight loss goal and importance of goal) and contrast outcomes between assessments that were made during temptations that did not lead to a lapse compared with temptations that led to a lapse. They found that use of these coping strategies were greater during temptations that did not lead to a lapse. Greater use of coping strategies also predicted lower temptation strength. An indirect relationship of using coping strategies on lapse occurrences via the temptation strength was also reported. Greater use of coping strategies reduced the strength of a temptation that subsequently reduced the likelihood of a lapse occurrence.

Chwyl et al.^22^ investigated how the strategies used in Carels et al.^21^ influenced whether a lapse would be reported on the current or next available assessment. They found that recently engaging in chores, cooking, spiritual activities, work/school tasks all decreased the likelihood that a lapse would be reported at the current assessment, whereas socializing increased the likelihood of a lapse being reported at the current assessment. Engaging in an indoor hobby, outdoor/exercise, or watching TV/social media had no effect on lapsing on the current assessment. Cooking and indoor hobbies increased the chances that a lapse was reported at the next available assessment.

Taken together, these findings suggest that engaging in coping strategies to manage the effects of the momentarily pressures that temptations have on adherence can prevent lapsing, possibly through reducing the strength of the temptation. There are many strategies that could be used in to cope, though certain activities may be more beneficial for preventing lapses.

### 3.5 Quality assessments

See section S.1.2 in the Supporting Information for the results of the modified NOS for EMA studies and CREMAS assessments. To summarize, most studies were of reasonable quality as assessed by the NOS, though all failed with regards to providing how sample sizes were determined or if any power analyses were performed. The quality of reporting for included studies was also found to be good, though there were many papers that did not report overall participant attrition rates or implement design features to address inherent issues of EMA such as participant burden or reactivity. Finally, none reported on the latency between receiving and completing random assessments. Overall, these findings suggest do not suffer from major methodological concerns, though the lack of power analyses reported means it is unclear to determine whether analyses were adequately powered to detect effects.

## 4 DISCUSSION

The aim of this review was to better understand the influences of real‐world experiences that are problematic for successful dietary adherence: Temptations and lapses. Our findings indicate that momentary fluctuations in appetite and affect during dieting precipitate as well as accompany these experiences. Employing strategies to cope with heightened sensations during temptations helps prevent lapsing. Abstinence‐violation effects occur following a lapse, and these are detrimental to self‐attitudes surrounding efficacy toward future dietary adherence. The temporal and contextually driven accounts in this review would be difficult to ascertain using conventional laboratory‐based or retrospective methods, which can only suggest the role of these processes in weight management.

Our findings were supportive of previous laboratory‐based and retrospective accounts of the barriers toward dieting.^13^, ^14^ Namely, increased levels of hunger, food cravings, and negative affect are detrimental for successful dietary adherence.^6^, ^7^, ^8^, ^11^ These factors influence the strength of temptations and reasons for lapsing.^10^, ^12^ Individuals who respond more negatively to lapses experience negative feelings of self‐efficacy and self‐regard.^15^

Coping strategies are thought to be important for both the control over eating and management of negative abstinence‐violation effects following a dietary lapse.^13^ While we found evidence for their use in temptations and shortly prior to lapsing,^21^, ^22^, ^28^ our review found no investigations of their use to manage negative abstinence‐violation effects (e.g., feelings the diet will not be successful), effects that can influence weight loss.^13^ Measuring coping strategies used to manage negative attitudes following lapsing could provide useful insight into maintaining dietary adherence after a violation in dieting goals.

Findings were inconsistent between analytical approaches (aims 1 and 2) for many appetitive and affective outcomes. One explanation could be that between‐study differences could influence the relationship between outcomes, temptations, and lapses. The studies in both aims 1 and 2 included self‐dieters and individuals at various stages in structured weight loss interventions. There are likely to be differences between dieters who diet on their own means and those who are enrolled in a structured weight loss program. However, due to a lack of primary investigations, this could not be fully explored with subgroup analyses using meta‐analysis.

Overall negative mood ratings differentiated temptations and lapses from random moments,^20^, ^21^ but evidence of effects on individual rating measures throughout the evidence for aims 1 and 2 were less consistent. One speculation is that individual measures of negative mood only assess one facet of the experience, while an overall mood score is a more general measure. The studies that examined the effect of the overall mood used composite scores composed of many individual mood ratings. These more holistic measures may be more sensitive to changes, whereas individual ratings are only sensitive to detecting changes in that specific sensation. This may be important as individuals who eat in response to strong emotions have a tendency to eat when experiencing specific emotions that have previously been paired with consumption.^37^ It may be the case that individual mood ratings are more predictive when examining effects at the case level (i.e., N‐of‐1 methods^38^). This is highly speculative and would require confirmation.

Another speculation is that individual ratings of negative mood are not particularly raised in the moments leading up to a lapse, but are heightened during a temptation and shortly following a lapse.^20^, ^21^, ^28^ A potential investigation could utilize both the prospective and contrast methods to measure differences between the moments precipitating and shortly following a lapse could shed further light into the temporal dynamic of affect and lapsing.

Our findings support the claim that individuals who struggle with achieving and maintaining weight loss have a poor range of appropriate coping strategies to deal with temptations.^13^ Managing temptations is a core component for regulatory control over eating.^10^ Strategies to increase the capacity to cope with these experiences could enhance dietary adherence. One approach is by monitoring changes in factors influential to dietary temptations and lapses and intervening when fluctuations are first detected (i.e., Just‐in‐time adaptive interventions; JITAI).

There has been some progress in the development of JITAI interventions.^25^, ^26^, ^32^, ^39^ JITAI for preventing dietary lapses have been associated with reductions in the amount of reported lapses and levels of weight loss though these claims require further validation.^25^, ^39^ There is currently an on‐going microrandomized trial into the effectiveness of different types of intervention delivered in moments of heightened risk for reducing the chance of lapsing within 2 h.^33^ It is important to note that all the evidence for JITAI effectiveness discussed here are iterations of the same intervention. More primary research using a diverse range of JITAI is required to determine the effectiveness of this for lapse prevention and weight loss.

Previous accounts claim that personalizing temptation management strategies based on key individual differences relating to reward‐responsivity and executive function should improve their effectiveness.^10^ Many investigations identified examined how between‐person levels influenced lapsing (e.g., do participants who report greater levels of hunger experience a greater likelihood of reporting a lapse?).^9^, ^22^, ^24^, ^27^, ^29^, ^30^, ^31^ These may be helpful in identifying individuals who may experience the greatest issue in managing appetite and affect during a negative energy balance. Dietary support may be tailored to inform bespoke coping strategies designed to manage the specific sensations that will likely pose the largest challenges for adherence.

Two main analytic approaches were taken in the studies within this review. A few studies^20^, ^21^, ^28^ compared user‐initiated assessments where a temptation or lapse has recently taken place to a random sample of assessments taken throughout the day. This is known as a contrast approach^18^ that compares the average levels of ratings for different experiences. A more common approach was to prospectively predict lapses by examining how within‐person changes prospectively predict the likelihood of an imminent lapse.^22^, ^24^, ^27^, ^29^, ^30^ These studies compare ratings on a given assessment to the individual's average of ratings and assess how differences influence the likelihood that a lapse is reported at the next available assessment (though two studies also reported on how factors influenced the likelihood for the current assessment^24^, ^29^). No studies have yet to examine the precedents of dietary temptations. More research could provide a more nuanced understanding of the dynamic interplay between temptations and lapses.

These approaches demonstrate some ways of examining the influences of real‐world experiences. A contrast approach collapses observations across time to characterize the exact moment of an event (i.e., during a dietary temptation or shortly following lapse), while prospective analyses investigate the dynamics in the moments leading up to the experience. For this reason, outcome measures are slightly different between these approaches. For example, lapse assessments using the contrast method are completed shortly following the occurrence, meaning a degree of retrospection is required (e.g., how hungry did you feel right before lapsing). Prospective analyses rely on random assessments that ask how they feel in when they are completing the rating.

Careful consideration of the appropriate outcome measures should be made when designing these studies as the decision will largely dictate which analysis approach can be taken. One study we identified used a different approach to those outlined above.^31^ They investigated the consequences of lapsing by using random assessments where participants were asked if they lapsed since completing the last assessment. This is similar to prospective designs, but instead they examined the consequences of lapsing which required a degree of retrospection (e.g., how did you feel when you last lapsed?). An issue here is that lapses may have taken place some hours prior to completing the rating. Using event‐based assessments (i.e., to complete shortly following a lapse) would be more appropriate for this type of design as they take place in the immediate moments surrounding the experience and limit the effect of any recall biases.^29^

### 4.1 Strengths and limitations

A strength of this systematic review is that it is the first comprehensive account of the current evidence from EMA studies of the impact of dieting on experiences of temptations and lapses in free‐living individuals. These findings build on those from previous retrospective and lab‐based approaches by shedding light onto the dynamics of these processes.

An additional strength is the emphasis on within‐person changes given these are a larger driver (relative to between‐person differences) in manipulations of energy balance.^17^ If weight loss is to be maximized across all individuals, then a shift of focus toward the specific characteristics which influence a person's likelihood of success is crucial.

Another strength is that this review highlighted how evidence yielded by different analytical approaches can be used alongside each other to add nuance in the understanding of the experiences. Using both approaches provides the ability to measure changes in both the precipitating and following moments of an event.

A major limitation was the lack of primary investigations. The few we identified were also from a diverse range of dieting approaches and stages of weight loss. For this reason, it was not possible to conduct meta‐analyses to determine reliable estimates of overall effects or subgroup differences. We instead created harvest plots to visualize the large array of finding and lack of substantiation behind most evidence, demonstrating that claims in this review are highly speculative.

A sufficiently powered meta‐analyses could provide an exploration of heterogeneity between studies to assess how methodological differences influence outcomes (e.g., type of EMA questions, assessment formats, populations used, and timepoints along weight loss attempt). The studies here demonstrate a range of differences relating to assessment strategies, analytic procedures, and stages/forms of weight loss interventions employed. These factors may explain some between‐study variation but can only be determined with more primary research so that meta‐analytic techniques would be suitably powered.

Another limitation is that some of the included studies employed paper‐and‐pencil diary methods of EMA.^20^, ^21^ This method is subject to bias through backfilling or hoarding of assessments given that time/date of completed entries cannot be verified. This demonstrates substantially lower levels of compliance than electronic assessments.^34^ Future EMA investigations should attempt to replicate these using electronic methods.

EMA has a heavy reliance on participants complying with the study protocol without supervision. This requires participants to correctly identify subjective states of interest (e.g., experiencing a dietary lapse) and report within the correct timeframe (e.g., within 30 min following the lapse). According to the results of the CREMAS checklist reported in supplementary analyses, most studies reported a procedure for training participants to the study protocol. However, none reported on the latency between receiving and completing random assessments. This concern does not include event‐based assessments where there is no objective way to validate reports. This is an inherent issue for event assessments, raising concerns for social desirability and non‐compliance. For example, participants may be reluctant to report if they experience a high frequency of lapses or they may simply forget to report. This does not apply to random assessments where it is validated through electronic time and date stamping. Financial incentives are commonly provided to complete as many random assessments as possible, though event‐based assessments cannot be incentivized.

Another concern with EMA is reactivity to experimental protocol.^40^ Bringing participants' awareness to their levels of hunger or negative mood may make these sensations more salient, which may have a knock‐on effect on subsequent behavior. For example, a participant may realize their level of hunger following an assessment, which may influence their eating behavior. The CREMAS assessment identified some studies included in the review that implemented measures of reactivity (e.g., “to what extent do you think the questions have an impact on your behaviour?”).^9^, ^21^, ^27^ Currently, there is no gold‐standard approach for addressing this methodological concern. Future EMA studies would benefit from guidelines toward implementing design features for addressing experimental reactivity.

## 5 CONCLUSION

EMA provides a richer account of the real‐world temporal dynamics that underlie temptations and lapses. The nuances in how these experiences unfold to determine real‐world dieting challenges would not be possible using laboratory or retrospective methods. This growing area of research demonstrates a huge potential to inform personalized dieting aids for use in high‐risk moments of lapsing or shortly following to ensure positive self‐attitudes remain high.

## CONFLICT OF INTEREST STATEMENT

ALA is funded by the Medical Research Council (MC_UU_00006/6) and was PI of an NIHR PGfaR‐funded study where the intervention was provided by WW (formerly Weight Watchers) at no cost. ALA was Treasurer for The Association for the Study of Obesity 2016‐2019. ALA is part of the WW Scientific Advisory Board, which the MRC Epidemiology Unit, University of Cambridge, is paid for their time. The University of Leeds has received payment for work by JCGH with Novo Nordisk, Dupont, Boehringer Ingelheim International GmbH, and Mars Wrigley. JCGH is the President of the European Association for the Study of Obesity (EASO). CAR has received grant funding from Unilever, and has acted as consultant to Boehringer Ingelheim, both of which are unrelated to the current work. PRC and JCGH have both received funding from the American Beverage Association.

## Supporting information


**Table S.1.2a** Quality assessment of studies using modified Newcastle‐Ottawa scales.
Table S.1.2b CREMAS checklist.
S.2 PRISMA Checklist.
S.1.1 Descriptions of quality assessments.
S.1.2 Results of quality assessments.

## Data Availability

The corresponding datafiles can be found on the Open Science Framework (osf.io/emwd9/).
