# Supplementary material for: A systematic review of ecological momentary assessment studies of appetite and affect in the experience of temptations and lapses during weight loss dieting
Source: Obes Rev. 2023 Jul 2;24(9):e13596. doi: 10.1111/obr.13596 (PMC10909537; doi:10.1111/obr.13596)
Supplement: Supplementary file 1 — Table S.1.2a Quality assessment of studies using modified Newcastle‐Ottawa scales. Table S.1.2b CREMAS checklist. S.2 PRISMA Checklist. S.1.1 Descriptions of quality assessments. S.1.2 Results of quality assessments. [file OBR-24-e13596-s001.pdf]

A systematic review of ecological momentary assessment studies of appetite and affect in the experience of temptations and lapses during weight loss dieting

Mark Randle [a] 🖂

Amy L Ahern [b]

Emma Boyland [c]

Paul Christiansen [c]

Jason C.G. Halford [c, d]

Jack Stevenson-Smith [c]

Carl Roberts [c]

Author information

[a] Cardiff University Brain Research Imaging Centre, Cardiff, United Kingdom

[b] MRC Epidemiology Unit, University of Cambridge, Cambridge, United Kingdom

[c] Department of Psychology, University of Liverpool, Liverpool, United Kingdom

[d] School of Psychology, University of Leeds, Leeds, United Kingdom

🖂Author for correspondence

Cardiff University Brain Research Imaging Centre, Maindy Road, Cardiff, CF24 4HQ

Email: Randlem@Cardiff.ac.uk

Supplementary materials

Contents

S.1.1 Descriptions of quality assessments………………..…………..……………..……Pg 2

S.1.2 Results of quality assessments……..………...……..……………………………..Pg 3

Table S.1.2a Quality assessment of studies using modified Newcastle-Ottawa scales .....Pg 6

Table S.1.2b CREMAS checklist………………………………………………….. .....Pg 7

S.2 PRISMA Checklist…………………………..………………….………..………....Pg 9

S.1.1 Descriptions of quality assessments

A modified Newcastle-Ottawa cohort scale adapted for cross sectional studies (NOS; Modesti et al., 2016)

We assessed quality of included studies using the Newcastle-Ottawa cohort scale adapted for cross sectional studies which rates quality of selection, comparability, and outcome (NOS for cross-sectional scale; Modesti et al., 2016). This was modified to assess the qualities of EMA observational investigations.

Selection is comprised of four items with a maximum score of five. These items assess sample representativeness, sample size, non-respondents and ascertainment of the exposure, all with a score of one except the latter item which has a maximum score of two. Items on sample representativeness were modified to assess representativeness of within-person assessments as these are the target of inference in EMA. A point was awarded for representativeness if RAs were utilised in the study design, and the time scheduling used for these assessments were either completely random times throughout the day (e.g. notified to perform an assessment at four random points throughout the day at any given time) or random timeframes (e.g. notified to complete between the hours of 8-10am, 10-12pm, and

12-4pm). A point was not awarded if RAs were not utilised or the time scheduling used for assessments were at fixed times (e.g. complete an assessment at 8am, 4pm, and 6pm).

Comparability is measured using two items with a maximum score of two. These items assess the most important factor for comparability (i.e. statistical analyses to account for clustering such as mixed models) as well as other important factors for comparability (i.e. controlling for differences in compliance and response rate during analyses, providing appropriate instructions to participants regarding assessment procedure and definitions of temptations and lapses).

Outcome has a maximum score of three and is measured by two items which assessed the type of assessment for the outcome used (maximum score of two) and appropriateness/description of statistical tests used (maximum score of one).

Checklist for reporting EMA studies (CREMAS; Liao et al., 2016)

An adapted STROBE checklist for EMA studies was used to assess the quality of reporting of the included studies. The STROBE is a commonly used checklist of items for observational studies. It contains 22 items that relate to the title, abstract, introduction, methods, results, and discussion sections of papers with the goal to improve the quality of reporting. Building on the STROBE checklist and the EMA design guidelines by Stone and Shiffman[27], a comprehensive checklist of specific items to be reported for EMA studies was also developed. In addition to STROBE checklist, additional methodological features, responses and compliance information is assessed with 16 items attaining to the following 5 main areas:

Sampling and measures: sample characteristics and tools used in the EMA protocol

Schedule: monitoring periods (number of waves from which data were collected), duration (number of days that each monitoring period lasted), prompt frequency (frequency of EMA prompts per day), and prompt interval (the time between each EMA prompt)

Technology and administration: use or lack of technology and method of administration of EMAs

Prompting strategy: methods used to cue participants—interval contingent (EMA prompts were set for certain intervals that were not random), random interval contingent (EMA prompts were set to be randomized throughout the day), event based (EMAs were recorded when eating occasions or physical activity occurred), or evening report (EMAs administered in the evenings to summarize the events of the day)

Response and compliance: participation rate, gathered data, missing data (i.e., unanswered and/or unprompted EMA surveys), latency (i.e., the time period between when participants receive an EMA prompt and when the EMA is answered), and attrition (i.e., the number of participants who dropped out of the study for any reason)

S.1.2 Results of quality assessments

Results for NOS for EMA (Table S.1.2a)

Most studies were of reasonable quality as assessed by the NOS. However, they uniformly performed poorly on sample size justification. No studies mentioned how sample sizes were determined or if power analyses were performed. If appropriate multilevel forms of analyses are employed to account for repeated measurements, large sample sizes are less of a problem than for other statistical approaches, as units of analyses are the within-person assessments and these are usually sufficiently powered. However, in multilevel analyses the major restricting factor is usually the group level sample size as these are usually lower than the number of within-person assessments, contain a greater amount of variation than within-person assessments, and have been shown to produce biased group-level estimates in smaller sample sizes McNeish & Stapleton (2016). Future investigations in this area should cite

guidelines that describe appropriate group-level sample sizes for multilevel modelling (Maas & Hox, 2015) to avoid scepticism surrounding sample sizes.

Most investigations used appropriate methods of analyses to account for multileveled datasets. Accounting for nesting of datapoints is important in repeated measures designs as within-person assessments are likely to be highly correlated which violates the assumption of independence of errors as datapoints. Most studies also reported appropriate statistics though a few failed to report confidence intervals for their associated *p*-values.

Reporting of differences between respondents and non-respondents or controlling for these in analyses was generally good in included studies, and most reported average compliance rates of EMA assessment protocol. Compliance with assessment protocol is a limitation of EMA as rates can have an impact on the statistical power of the study, particularly if data are missing not at random and are systematic (e.g. missing prompts due to working hours) (Graham, 2009). There is currently no ‘gold-standard’ rate of compliance, though a rule of thumb is that compliance rates of at least 80% are considered acceptable (Jones et al., 2019). Providing descriptive information on compliance rates is essential as it may indicate whether a particular EMA assessment procedure may be too burdensome and allows for reviews to be conducted that examine overall compliance rates across studies as well as predictors that may influence compliance that could be used to facilitate higher rates (Jones et al., 2019).

All studies used subjective measures and self-report which are associated with information bias such as socially desirability and demand characteristics. Furthermore, one problem of EMA investigations relate to reactivity to experimental procedure which could also introduce bias into measures (Rowan et al., 2007). However, given the subjective nature of appetite ratings these experiences would be difficult to measure otherwise. Future investigations could combine both free-living and laboratory-based approaches to validate changes in average

levels of real-world subjective appetite ratings such as hunger throughout weight loss with physiological markers.

Results for CREMAS (Table S.1.2b)

Most (7/10) studies identified the paper as an EMA study in the title, however all performed better providing a rationale for EMA in the introduction (10/10).

Regarding methodology, only three studies (7/10) did not report (or refer to a primary study which detailed) how participants were trained in the EMA protocol. All (10/10) papers referred to the technology which was used to administer EMA, and 9 out of 10 papers referred to the wave duration (i.e. 1 period of EMA) and all studies reported the number of days each wave lasted. All studies reported the type of prompts utilised, and 9 out of 10 studies reported how many random prompts were sent per day (if the study utilised this type of prompt). 4 out of 10 studies utilised a design feature to control for EMA methodological limitations such as reactivity or participant burden. 4 out of 10 studies reported on the participant attrition. All but one study reported on the results of the prompt delivery (e.g. how many random prompts were received/events were reported). No studies reported on the latency between receiving a prompt and reporting of the prompt. 7/10 reported on the compliance rate of EMA prompts, and 4 out of 10 studies reported on whether missing data was related to time or demographic-related variables.

Regarding discussion points, 6 out of 10 studies reported the limitations of the study in light of using an EMA methodology, and all studies discussed the benefits of using EMA and how it helped achieved the desired aims of the paper.

Table S.1.2a – Quality assessment of studies using modified Newcastle-Ottawa scales for assessing included studies of appetite measures with EMA during ER

**Study***	**Selection**				**Comparability**	**Outcome**		**Total (max 9●)**
	Representiveness of sample (●)	Sample size (●)	Non-respondents (●)	Ascertainment of the exposure (●●)	(●●)	Assessment of the outcome (●)	Statistical test (●)	
Carels et al. (2001)	●	-	-	●●	●●	●	-	●●●●●● (6)
Carels et al. (2004)	●	-	-	●●	●●	●	-	●●●●●● (6)
Chwyl et al. (2022)	●	-	●	●●	●●	●	●	●●●●●●●● (8)
Crochiere et al. (2022)	●	-	-	●●	-●	●	●	●●●●●● (6)
Forman et al. (2017)	●	-	●	●●	●●	●	●	●●●●●●●● (8)
Goldstein et al. (2018)	●	-	●	●●	●●	●	●	●●●●●●●● (8)
McKee et al. (2014)	●	-	-	●●	●-	●	-	●●●●● (5)
Sala et al. (2021)	●	-	-	●●	●●	●	●	●●●●●●● (7)
Schumacher et al. (2018)	●	-	●	●●	●●	●	●	●●●●●●●● (8)
Thøgersen-Ntoumani et al. (2021)	●	-	●	●●	●●	●	●	●●●●●●●● (8)

Topic	Item	Checklist item	Carels et al 2001 Pg no.	Carels et al 2004a Pg no.	Chwyl et al. (2022) Pg no.	Crochiere et al. (2021) Pg no.	Forman et al. (2017) Pg no.	Goldstein et al. (2018b) Pg no.	McKee et al. (2014) Pg no.	Sala et al. (2021) Pg no.	Schumacher et al. (2018) Pg no.	Thøgersen-Ntoumani et al. (2021) Pg no.
**Title**	1	Include ecological momentary assessment in title and key words	1	1	1	-	1	1	1	-	1	-
**Introduction**												
Rationale	2	Briefly introduce the concept of EMA and provide reasons for utilizing EMA for this study or topic of interests (eg, to examine time-varying predictors of unhealthy eating occasions in children’s daily lives)	3	2	1-2	2	2	2	2	2-3	2	4
**Methods***												
Training	3	Indicate if, and by what methods, training of participants for EMA protocol was used.	9	2	3	-	4	*	4	4-5	*	6
Technology	4	Describe what technology, if any, was used. Include the following information: device (eg, mobile phone, portable computer), model (eg, Nexus 4, iPod), operating system (eg, Android, Windows), and EMA program name	3	2	3	3	4	3	3	4-5	*	6
Wave duration	5	State the number of waves for the study (eg, 2 monitoring periods over the course of 1 year)	9	2	3	-	4	3	3	4-5	2	6
Monitoring period	6	State the number of days each wave of the study lasted, and how many weekdays versus weekend days	9	2	3	3	4	3	3	4-5	2	6
Prompting design	7	Indicate the prompting strategy used for the study (eg, event-based, interval-based, or a combination of the two). If using interval-based strategy, indicate what type of schedule is used (eg, fixed, random, or hybrid interval)	9	2	3	3	4	3	4	5	2	6
Prompt frequency	8	Intended frequency of prompts per day. Break down by weekdays and weekend days if applicable	-	3	3	-	4	3	NA	5	2	6
Design features	9	Describe any design feature to address potential sources of bias (eg, reactivity) or participant burden (eg, EMA questions appearing in different orders)	-	3	-	-	4	3	6	-	*	-
**Results***												
Attrition	10	Indicate participant attrition throughout the study; report attrition rates both by monitoring days and waves, if applicable	-	-	-	4	5	3	-	-	*	9 – 10
Prompt delivery	11	Report number of EMA prompts that were planned to be delivered. If possible, also report the number of EMA prompts that were actually received by participants and indicate reasons for why prompts were not sent out (eg, technical issues or participant noncompliance reason such as phone was powered off)	-	3	4	4	5	4	NA	6	*	9
Latency	12	Report the amount of time from prompt signal to answering of prompt	-	-	-	-	-	-	NA	-	-	-
Compliance rate	13	Report total answered EMA prompts across all subjects and the average number of EMA prompts answered per person. Report compliance rate both by monitoring days and waves, if applicable. Indicate reasons for noncompliance, if known	-	3	4	-	5	4	5	-	3	9

Missing data	14	Report whether EMA compliance is related to demographic or time-varying variables	-	-	-	-	5	4	NA	7	3	-
**Discussion**												
Limitations	15	Discuss limitations of the study, taking into account sources of potential bias when using EMA methods (eg, reactivity, use of technology)	-	7/8	7	-	11	6 to 8	-	10	-	17
Conclusions	16	Provide a general interpretation of results and discuss the benefits of using EMA (eg, improving understanding of daily behaviors)	14/15	8	7	5	11/12	8	9	10	4	17

S.5 PRISMA 2009 Checklist

Section/topic	#	Checklist item	Reported on page #
**TITLE**			
Title	1	Identify the report as a systematic review, meta-analysis, or both.	2
**ABSTRACT**			
Structured summary	2	Provide a structured summary including, as applicable: background; objectives; data sources; study eligibility criteria, participants, and interventions; study appraisal and synthesis methods; results; limitations; conclusions and implications of key findings; systematic review registration number.	2
**INTRODUCTION**			
Rationale	3	Describe the rationale for the review in the context of what is already known.	3
Objectives	4	Provide an explicit statement of questions being addressed with reference to participants, interventions, comparisons, outcomes, and study design (PICOS).	4
**METHODS**			
Protocol and registration	5	Indicate if a review protocol exists, if and where it can be accessed (e.g., Web address), and, if available, provide registration information including registration number.	5
Eligibility criteria	6	Specify study characteristics (e.g., PICOS, length of follow-up) and report characteristics (e.g., years considered, language, publication status) used as criteria for eligibility, giving rationale.	5-6
Information sources	7	Describe all information sources (e.g., databases with dates of coverage, contact with study authors to identify additional studies) in the search and date last searched.	5
Search	8	Present full electronic search strategy for at least one database, including any limits used, such that it could be repeated.	5
Study selection	9	State the process for selecting studies (i.e., screening, eligibility, included in systematic review, and, if applicable, included in the meta-analysis).	5-6

Data collection process	10	Describe method of data extraction from reports (e.g., piloted forms, independently, in duplicate) and any processes for obtaining and confirming data from investigators.	6
Data items	11	List and define all variables for which data were sought (e.g., PICOS, funding sources) and any assumptions and simplifications made.	6
Risk of bias in individual studies	12	Describe methods used for assessing risk of bias of individual studies (including specification of whether this was done at the study or outcome level), and how this information is to be used in any data synthesis.	7
Summary measures	13	State the principal summary measures (e.g., risk ratio, difference in means).	NA
Synthesis of results	14	Describe the methods of handling data and combining results of studies, if done, including measures of consistency (e.g., I^2) for each meta-analysis.	8

PRISMA 2009 Checklist

Section/topic	#	Checklist item	Reported on page #
Risk of bias across studies	15	Specify any assessment of risk of bias that may affect the cumulative evidence (e.g., publication bias, selective reporting within studies).	None
Additional analyses	16	Describe methods of additional analyses (e.g., sensitivity or subgroup analyses, meta-regression), if done, indicating which were pre-specified.	N/A
**RESULTS**			
Study selection	17	Give numbers of studies screened, assessed for eligibility, and included in the review, with reasons for exclusions at each stage, ideally with a flow diagram.	Figure 1
Study characteristics	18	For each study, present characteristics for which data were extracted (e.g., study size, PICOS, follow-up period) and provide the citations.	Table 1
Risk of bias within studies	19	Present data on risk of bias of each study and, if available, any outcome level assessment (see item 12).	supplementary

Results of individual studies	20	For all outcomes considered (benefits or harms), present, for each study: (a) simple summary data for each intervention group (b) effect estimates and confidence intervals, ideally with a forest plot.	8 – 13
Synthesis of results	21	Present results of each meta-analysis done, including confidence intervals and measures of consistency.	8 – 13
Risk of bias across studies	22	Present results of any assessment of risk of bias across studies (see Item 15).	None
Additional analysis	23	Give results of additional analyses, if done (e.g., sensitivity or subgroup analyses, meta-regression [see Item 16]).	None
**DISCUSSION**			
Summary of evidence	24	Summarize the main findings including the strength of evidence for each main outcome; consider their relevance to key groups (e.g., healthcare providers, users, and policy makers).	13 – 21
Limitations	25	Discuss limitations at study and outcome level (e.g., risk of bias), and at review-level (e.g., incomplete retrieval of identified research, reporting bias).	18 – 20
Conclusions	26	Provide a general interpretation of the results in the context of other evidence, and implications for future research.	20 - 21
**FUNDING**			
Funding	27	Describe sources of funding for the systematic review and other support (e.g., supply of data); role of funders for the systematic review.	End section

From: Moher D, Liberati A, Tetzlaff J, Altman DG, The PRISMA Group (2009). Preferred Reporting Items for Systematic Reviews and Meta-Analyses: The PRISMA Statement. PLoS Med 6(7): e1000097.
doi:10.1371/journal.pmed1000097

For more information, visit: **www.prisma-statement.org**.

Page 2 of 2

References

Modesti, P. A., Reboldi, G., Cappuccio, F. P., Agyemang, C., Remuzzi, G., Rapi, S., et al. (2016). Panethnic Differences in Blood Pressure in Europe: A Systematic Review and Meta-Analysis. *PloS One*, *11*(1), e0147601. https://doi.org/10.1371/journal.pone.0147601

Liao, Y., Skelton, K., Dunton, G., & Bruening, M. (2016). A Systematic Review of Methods and Procedures Used in Ecological Momentary Assessments of Diet and Physical Activity Research in Youth: An Adapted STROBE Checklist for Reporting EMA Studies (CREMAS). *Journal of medical Internet research*, *18*(6), e151. https://doi.org/10.2196/jmir.4954

McNeish, D. M., & Stapleton, L. M. (2016). The Effect of Small Sample Size on Two-Level Model Estimates: A Review and Illustration. *Educational Psychology Review*, *28*(2), 295–314. https://doi.org/10.1007/s10648-014-9287-x

Maas, C. J. M., & Hox, J. J. (2015). Sufficient Sample Sizes for Multilevel Modeling. *Methodology*, *1*(3), 86–92. doi:10.1027/1614-1881.1.3.86

Graham, J. W. (2009). Missing Data Analysis: Making It Work in the Real World. *Annual Review of Psychology*, *60*(1), 549–576. https://doi.org/10.1146/annurev.psych.58.110405.085530

Jones, Andrew, Remmerswaal, D., Verveer, I., Robinson, E., Franken, I. H. A., Wen, C. K. F., & Field, M. (2019). Compliance with ecological momentary assessment protocols in substance users: a meta-analysis. *Addiction*, *114*(4), 609–619. https://doi.org/10.1111/add.14503

Rowan, P. J., Cofta-Woerpel, L., Mazas, C. A., Vidrine, J. I., Reitzel, L. R., Cinciripini, P. M., & Wetter, D. W. (2007). Evaluating Reactivity to Ecological Momentary Assessment During Smoking Cessation. *Experimental and Clinical Psychopharmacology*, *15*(4), 382–389. https://doi.org/10.1037/1064-1297.15.4.382
